# Anesthetic considerations for cesarean section in a parturient complicated by Scimitar syndrome-like pathophysiology

**DOI:** 10.1186/s40981-018-0215-9

**Published:** 2018-11-09

**Authors:** Satoshi Kurokawa, Keiko Hirooka, Mirei Nagai, Makoto Ozaki, Minoru Nomura

**Affiliations:** Faculty of Medicine, Department of Anesthesiology, Tokyo Women’s Medical University, Kawada-cho 8-1, Shinjuku-ku, Tokyo, Japan

**Keywords:** Lung hypoplasia, Pulmonary venous atresia, Scimitar syndrome, Impaired respiratory function, Cesarean section, General anesthesia

## Abstract

**Background:**

Pre-existing poor respiratory function is a significant challenge for women to successfully continue pregnancy and accomplish delivery.

**Case:**

Pregnancy and delivery were successfully managed without any maternal or neonatal complications, in a 26-year-old woman with severely impaired respiratory function, due to a unilateral hypoplastic lung, accompanying Scimitar syndrome-like circulation. Hyperventilation, normally observed even at the first trimester, was absent by the end of the second trimester. This would indicate her ventilation must have reached utmost capacity. Premature delivery by the mode of elective cesarean section delivery was, therefore, the most reasonable option. General anesthesia, combined with a continuous epidural infusion of low-concentrate local anesthetics, containing opioid, was sufficient to avoid the need for unexpected mechanical ventilation in intra- and early postoperative periods and to provide excellent post-partum analgesia.

**Conclusion:**

This combination can be a potent alternative in tailoring anesthesia for cesarean section in women with extremely impaired pulmonary reserve.

## Introduction

Pre-existing poor respiratory function is a significant challenge for women to successfully continue pregnancy and accomplish delivery. We describe a patient with severely impaired pulmonary function, somewhat resembling Scimitar syndrome, who presented premature delivery, in the manner of cesarean section. Written informed consent for this presentation was obtained from the patient.

## Case

A 26-year-old woman with complicated cardio-pulmonary anomaly became pregnant. In early infancy, bilateral hypoplasia of the lung, total atresia of left pulmonary veins (PVs), hypoplastic left branch pulmonary artery (PA), and anomalous aorto-pulmonary collateral to the left lung had been detected at another hospital. The trans-catheter occlusion of the collateral was, subsequently, conducted immediately after the detection. Thereafter, she was followed without any other trans-catheter or surgical interventions. An angiography performed at 5 years of age confirmed that the left PA was totally occluded and that the bronchial artery, substituting for PA, perfused the left lung.

**Fig. 1:**
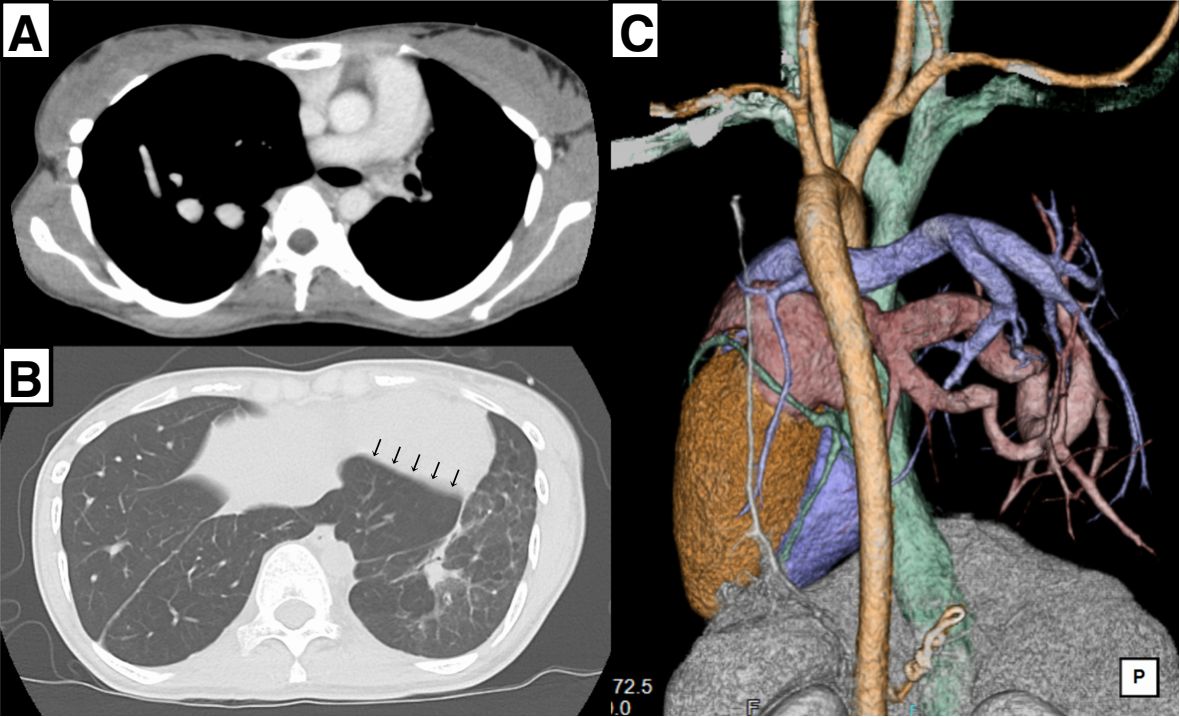
**a** Left pulmonary artery (PA) branching from main PA was absent. **b** Right lung partially invaded into left thorax (*arrows*), and left lung contained both fibrous and bullous lesions. **c** Three-dimensional reconstruction image of the posterior aspect of the heart and major vessels indicated that neither PA (*blue*) nor pulmonary vein (PV) (*red*) was present in the left lung, while marked dilations of both PA and PV were seen in the right lung

Prior to conception, she had undergone several examinations. A computed tomography scan revealed fibrosis, bullous changes, and poorly developed airways in the small left lung and an over-distended right lung partially invading the left thorax (Fig. 1). Cardiopulmonary exercise testing indicated severely lowered exercise capacity and severely impaired ventilatory efficiency. It also revealed a significant decrease in SpO_2_ to a nadir of 92% from a baseline of 97% at rest. Her forced vital capacity (FVC) was restrictive at 1340 ml (45.4% of predicted), and forced expiratory volume in 1 s (FEV_1.0_) was also reduced to 860 ml (32.3% of predicted). Although she had no complaints of respiratory symptoms, forced respiration with effort was clearly observed and emaciation was significant. Her weight was only 31.9 kg (body mass index 15.0 kg/m^2^) before conception.

**Fig. 2:**
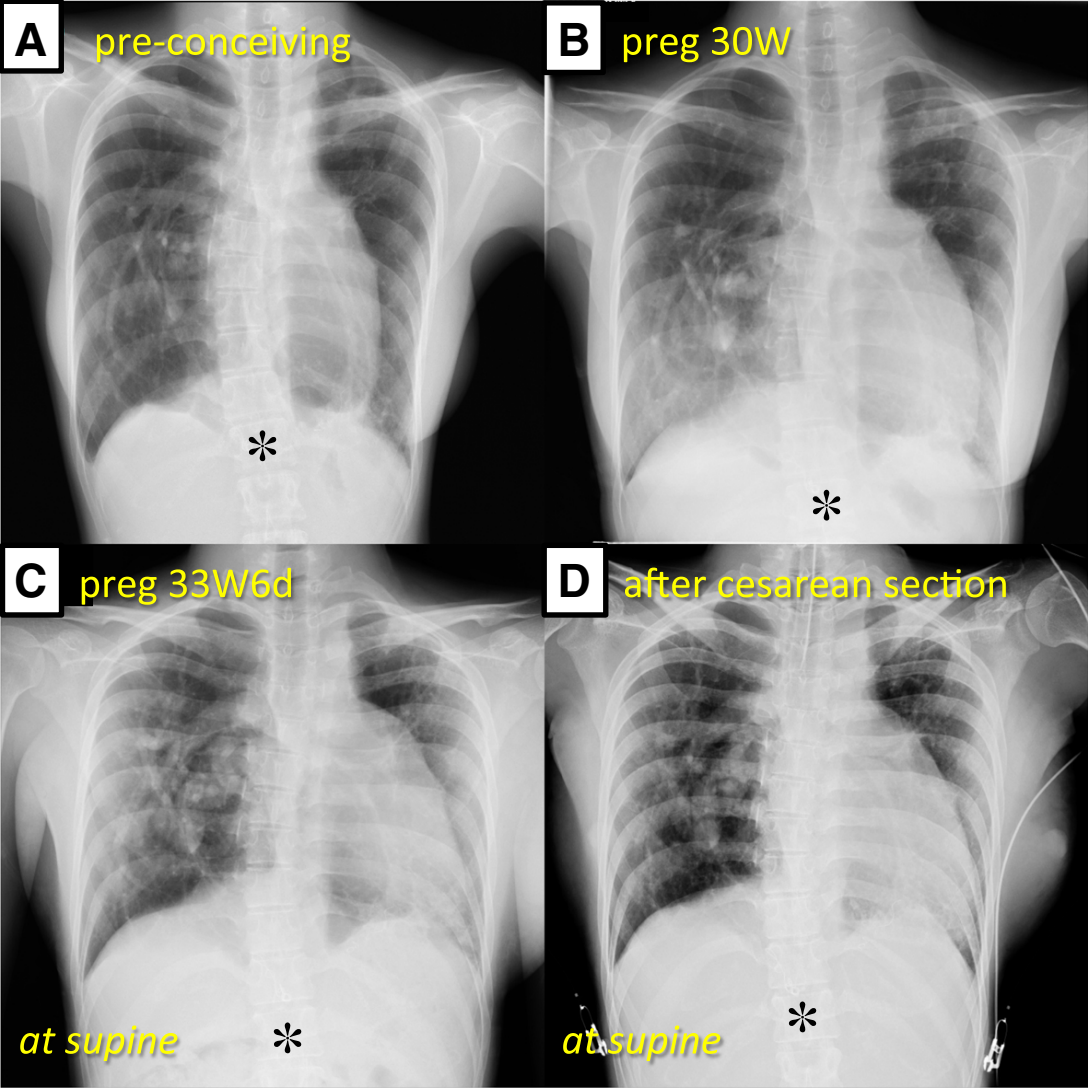
**a** Chest X-ray, at pre-conception, demonstrated that the right lung looked over-infiltrated and was bulging into the left thorax. **b** At 30 weeks of gestation, the position of the diaphragm was located at the same level as before conception, while vascular shadows in the right lung field were increased. (Asterisk marks the 12th thoracic vertebra in each chest X-ray.) **c** On the day before cesarean section, chest X-ray in supine position confirmed no significant displacement of the diaphragm, when considering the impact of the posture on diaphragm position; however, it revealed further increase of vascular shadows in the right lung field. **d** No abnormal findings in the lung field and no significant change of the diaphragm level, compared with those the day before the operation, were confirmed, immediately after the cesarean section

During the progress of her gestation, she presented neither deterioration of respiratory symptoms subjectively nor any event requiring respiratory treatment. The spirometry conducted at 27 weeks of gestation revealed no significant change in the measurements. According to a chest X-ray, no apparent change in the lung field was observed; furthermore, the height of her diaphragm was unchanged at the same vertebral level, without any elevation, even at 30 weeks of gestation, as compared with before conception (Fig. 2). Moreover, transthoracic echocardiography repeatedly performed in her second and third trimesters confirmed absence of significant pulmonary hypertension and well-maintained right ventricular function. Blood gas analysis (BGA) repeatedly conducted every week after 27 weeks of gestation revealed that both arterial carbon dioxide tension (36–43 mmHg) and bicarbonate ion (23.0–27.0 mEq/L) were still unchanged, with normal values quite similar to non-pregnant state, even after the end of the second trimester.

It could be speculated that her respiration did not have enough reserve to tolerate any further increase in oxygen demand and had already reached the threshold to induce catastrophic deterioration at any moment, based on the following points. First, BGA data indicated she had not had any ventilatory reserve, to produce the hyperventilation state normally observed, even since the first trimester. Second, a possible elevation of the diaphragm, in the remaining gestation period, must have made a critical mismatch of ventilation for oxygen demand. And finally, her effort respiration had been continued. To avoid possible catastrophic deterioration of respiration, in the latter period of the third trimester, therefore, an elective cesarean section at exactly 34 weeks was planned.

We chose a general anesthesia (GA) combined with epidural catheterization at Th11/12. Rapid sequence induction with thiopental of 200 mg and rocuronium of 40 mg was followed by tracheal intubation. The baby weighing 2011 g was delivered at 5 min after the induction of GA (Apgar score 7/8). Then, the GA was maintained with a combination of sevoflurane of 1–1.5%, remifentanil infusion at 0.3 μg/kg/min, and additional bolus infusion of fentanyl up to 200 μg. The continuous infusion of 0.1% levobupivacaine containing fentanyl of 2 μg/ml at 4 ml/h was commenced immediately after the delivery of the baby. Spontaneous respiration was fully recovered soon after the operation, and early extubation was accomplished in the operating room, after confirming the absence of both abnormal findings on chest X-ray (Fig. 2) and derangement of BGA. She did not complain of any respiratory symptoms and had stable respiration and no post-operative pain, throughout the period of continued epidural analgesia. Her post-operative course was uneventful, and she was discharged on the 11th post-operative day.

## Discussion

Scimitar syndrome is characterized by a combination of a partial anomalous PV return into the inferior vena cava (IVC), lung hypoplasia, and systemic PA supply, located in the ipsilateral lung to IVC. Additionally, the syndrome is occasionally accompanied by dextrocardia. Clinical features of the syndrome vary widely among the cases. Some patients present shortness of breath, dyspnea, or other respiratory symptoms to various extents of severity; in contrast, some cases are asymptomatic. Other deliveries by women with Scimitar syndrome have been distinctly reported [1, 2]. However, no data on pulmonary function has been available to consider the threshold for predicting premature deliveries, or the need for employing cesarean section as the mode of delivery in patients with Scimitar syndrome. Given the unique circulation features of this syndrome, in which a hypoplastic lung perfused by the systemic arterial supply is consequently isolated from pulmonary circulation, in addition to restrictive pulmonary function, it is speculated that the threshold may be markedly larger than those in other types of pulmonary disease. Cystic fibrosis (CF) induces impaired clearance and obstruction of viscous secretions in the respiratory tract, resulting in repeated pulmonary infections and progressive mixed respiratory dysfunction. A recent retrospective study in the UK demonstrated that CF women with FEV_1.0_ ≦ 60% were more likely to have premature delivery, typically by cesarean section [3]. In isolated restrictive disorders, based on a study by Lapinsky et al., women with FVC < 40% are likely to deliver before reaching full-term, usually by cesarean section [4]. Our case had FEV_1.0_ and FVC similar to the abovementioned thresholds reported in CF and restrictive lung disorders, respectively, besides Scimitar syndrome-like lung perfusion.

We considered the carbon dioxide tension in BGA to have been a reliable key to precisely comprehend the maternal respiratory condition and reserve. The tension must, inevitably be low, at approximately 28–30 mmHg, even in the first trimester for healthy pregnant women [5]. Given the normal tension even after the second trimester, her ventilation must have already reached a plateau at the maximum level and also have been restricted at a level significantly lower than the increased ventilation normally occurring in pregnancy. Based on these factors, choosing cesarean section, even though premature, for the present case, was quite reasonable.

In most cases of cesarean section in patients who have apparently impaired pulmonary function, neuraxial blocks have been used to avoid maternal endotracheal intubation, followed by possible prolonged mechanical ventilation [4, 6, 7]. Muammer et al. reported that spinal anesthesia (SA) for cesarean section had no negative effects, on both FVC and FEV_1.0_, even in a woman with CF, who had severely impaired pulmonary function [7]. GA could be, however, preferred from the vantage point of preventing respiratory muscle paralysis reaching the high thoracic level, which can be observed concomitant with neuraxial blocks alone. Ishihara investigated the impact of spinal or epidural anesthesia alone on both FVC and FEV_1.0_ [8]. Analysis of 77 SA cases demonstrated that the reduction of FVC began when the maximum sensory block reached Th9 and that FVC further declined linearly to 59.1% of the baseline value at the measurement before conducting anesthesia, in a negative correlation with the elevation of the sensory block level to C8. In 157 cases of epidural anesthesia analyzed, the linear decline of FVC, starting at the maximum sensory block level of Th7 and reaching 70.9% of the baseline value at the block level of C8, similar but less steep than that found in SA, was also observed. Both anesthetic techniques, however, appeared to have no impact on FEV_1.0_. Nowadays, combined spinal-epidural anesthesia (CSEA) or low-dose SA has been favored for elective cesarean delivery [9–13]. No matter which CSEA or SA technique we choose, however, the maximum sensory block must extend to the high thoracic vertebral level for cesarean section. Teoh et al. showed that CSEA, even when using ultra-low dose bupivacaine of 3.75 mg, followed by a 3-ml epidural infusion of 1.5% lidocaine, provided the maximum sensory block reaching to a median level of Th3 (Th2–Th6), although significantly lower than the level of Th2 (C2–Th6) observed in the conventional dose (9 mg) group [13]. This implies CSEA can paralyze respiratory muscle to a significant extent which may induce possible respiratory deterioration in a case which has severely impaired respiratory function, irrespective of the intrathecal bupivacaine dose. Postoperative analgesia should also be considered an important component to successfully manage postpartum respiration in patients with reduced pulmonary reserve. We, therefore, chose GA combined with continuous epidural anesthesia for postoperative analgesia, using low-concentrated local anesthetics containing opioid, primarily to avoid thoracic muscle paralysis.

In summary, our successful management of the present case demonstrates this combination is very useful in tailoring anesthesia for cesarean section, in the cases with extremely impaired pulmonary reserve.

## Abbreviations

BGA: Blood gas analysis
CF: Cystic fibrosis
CSEA: Combined spinal-epidural anesthesia
FEV_1.0_: Forced expiratory volume in 1 s
FVC: Forced vital capacity
GA: General anesthesia
IVC: Inferior vena cava
PA: Pulmonary artery
PV: Pulmonary vein
SA: Spinal anesthesia
SpO_2_: Peripheral capillary hemoglobin oxygen saturation
